# Subtyping of type 2 diabetes from a large Middle Eastern biobank: Implications for precision medicine

**DOI:** 10.1016/j.molmet.2025.102195

**Published:** 2025-06-23

**Authors:** Nayra M. Al-Thani, Shaza B. Zaghlool, Salman M. Toor, Abdul Badi Abou-Samra, Karsten Suhre, Omar M.E. Albagha

**Affiliations:** 1 College of Health and Life Sciences (CHLS), Hamad Bin Khalifa University (HBKU), Qatar Foundation (QF), P.O. Box 34110, Doha, Qatar; 2 Bioinformatics Core, Weill Cornell Medicine-Qatar, Education City, 24144, Doha, Qatar; 3 Qatar Metabolic Institute, Hamad Medical Corporation, P.O. Box 3050, Doha, Qatar; 4 Englander Institute for Precision Medicine, Weill Cornell Medicine, New York, NY, 10021, USA; 5 Qatar Biomedical Research Institute (QBRI), Hamad Bin Khalifa University (HBKU), Qatar Foundation (QF), P.O. Box 34110, Doha, Qatar

**Keywords:** Type 2 diabetes, SIDD, SIRD, MOD, MARD, MODY, CKD, MASLD

## Abstract

Type 2 diabetes (T2D) can be classified into Severe Insulin-Deficient Diabetes (SIDD), Severe Insulin-Resistant Diabetes (SIRD), Mild Obesity-related Diabetes (MOD), and Mild Age-related Diabetes (MARD). This classification can help in predicting disease complications and determining the best treatment for individuals. However, the applicability of this classification to non-European populations and sensitivity to confounding factors remain unclear. We applied k-means clustering to a large Middle Eastern biobank cohort (Qatar Biobank; QBB, comprising 13,808 individuals; 2,687 with T2D). We evaluated the efficacy of the European cluster coordinates and analyzed the impact of using actual age on clustering outcomes. We examined sex differences, analyzed insulin treatment frequency, investigated the clustering of monogenic diabetes (MD) focusing on maturity-onset diabetes of the young (MODY), and evaluated the prevalence of chronic kidney disease (CKD) and Metabolic Dysfunction-Associated Steatotic Liver Disease (MASLD) among T2D subtypes. We identified the four T2D subtypes within a large Arab cohort. Data-derived centers outperformed European coordinates in classifying T2D. The use of actual age, as opposed to age of diagnosis, impacted MOD and MARD classification. Obesity prevalence was significantly higher in females but it did not translate to worse disease severity, as indicated by comparable levels of HbA1C and HOMA2-IR. Insulin was predominantly prescribed to individuals in SIDD and SIRD. SIRD displayed the highest risk of CKD and MASLD, followed by MOD and SIDD compared to MARD. Interestingly, most MODY individuals were clustered within MARD, further highlighting the need for precise classification and tailored interventions. The observed sex differences underscore the importance of tailoring treatment plans for females compared to males. Individuals who are at a higher risk of CKD and MASLD may require closer monitoring and physician oversight. Additionally, in populations without access to genetic testing, likely MODY individuals can be identified within the MARD cluster. Our findings strongly support the need for a transition to more personalized, data-driven treatment approaches to minimize diabetes-related complications and improve disease outcomes.

## Highlights

• We successfully classified 2,687 individuals with T2D into four distinct subtypes (SIDD, SIRD, MOD, MARD).
• Obesity was significantly higher among females; however, this did not increase disease severity, as shown by similar levels of HbA1C and HOMA2-IR.
• The prevalence of Chronic Kidney Disease (CKD) and Metabolic dysfunction–associated steatotic liver disease (MASLD) was highest in SIRD, followed by SIDD and MOD.
• MODY individuals can be identified within the MARD cluster in populations without access to genetic testing.

## 1 Introduction

The International Diabetes Federation predicts that by 2030, over 600 million individuals will be living with diabetes [1]. Type 2 diabetes (T2D) is characterized by dysregulation of beta-cell function and insulin resistance, posing an increased risk for complications such as cardiovascular disease and renal failure [2,3]. Traditional classification systems do not facilitate individualized treatment for T2D patients. In 2018, a novel scheme proposed categorizing T2D into four subtypes based on six measured variables: Hemoglobin A1C (HbA1C) levels, body mass index (BMI), age of diagnosis, presence of glutamate decarboxylase antibodies (GADA), homeostasis model assessment (HOMA) 2 of estimating beta-cell function (HOMA2-%B) and insulin resistance (HOMA2-IR) [4]. The T2D diabetes classification included Severe Insulin-Deficient Diabetes (SIDD), Severe Insulin-Resistant Diabetes (SIRD), Mild Obesity-related Diabetes (MOD), and Mild Age-Related Diabetes (MARD) [4]. Type 1 diabetes (T1D) was identified as GADA positive Severe Autoimmune Diabetes (SAID) subtype. This framework, which was developed using the Swedish All New Diabetics in Scania (ANDIS) cohort, has gained traction and offers valuable insights into potential complications associated with T2D [[5], [6], [7], [8], [9], [10]], and have been investigated in more than 50 studies, reviewed recently [11]. Classifying T2D individuals facilitates a precision medicine approach, allowing for tailored interventions to potentially slow the progression of complications.

Recently, we applied clustering to the Qatar Biobank (QBB) cohort (n = 6,218), revealing unique proteomic and metabolomic signatures among the identified subtypes [12]. Notably, SIDD individuals exhibited autoimmune features linked to complement system activation, while SIRD individuals displayed impaired insulin signaling. MOD individuals showed elevated levels of fatty acid-binding protein and leptin, and individuals classified as MARD exhibited the healthiest profiles [12].

Numerous studies suggest that subtype clustering is dynamic, that individuals potentially shift between clusters over time, and show varying outcomes across different populations [9]. For instance, research in the Indian population replicated SIDD and MARD subtypes but also identified two novel clusters [5]. In the Japanese population, individuals classified as SIRD were diagnosed at a younger age and had the highest BMI, a pattern not observed in the MOD subtype [8]. Insulin therapy is commonly prescribed for those with severe HbA1C levels, while in recent findings, usage of insulin was higher in SIDD, SIRD, and MARD subtypes [8]. Moreover, individuals with monogenic diabetes (MD), such as those with maturity-onset diabetes of the young (MODY), are often misclassified as having T2D, as previously demonstrated in the QBB cohort [13].

It has been reported previously that chronic kidney disease (CKD) risk is more prevalent in SIRD [4,14,15]. Studies reported that SIRD, SIDD, and MOD had a higher prevalence [14], while another study reported the prevalence was higher in SIDD and MARD [15]. The inconsistent results of CKD risk across subtypes suggest that classification can define individuals with a higher risk of CKD in the severe clusters (SIDD and SIRD) but not the mild clusters (MOD and MARD). Moreover, Nonalcoholic Fatty Liver Disease (NAFLD), recently refined to Metabolic Dysfunction-Associated Steatotic Liver Disease (MASLD) to underpin the association with metabolic dysfunction observed in T2D and hepatic steatosis (fatty liver) [16], was more prevalent in SIRD in the ANDIS cohort [4] and the Mexican cohort [17] but higher in MOD in the Chinese cohort [18].

In this study, we applied the Ahlqvist et al. [4] clustering scheme to an extended QBB cohort comprising 13,808 individuals. Our analysis evaluated the applicability of European-derived coordinates, investigated the influence of using actual age versus age at diagnosis on T2D classification, assessed the effect of diabetes duration on clustering, and examined sex-based differences across subtypes within the cohort. Furthermore, we analyzed insulin prescription patterns among T2D subtypes, explored the clustering of MD focusing on MODY individuals, and assessed CKD and MASLD risk across the QBB cohort.

## 2 Results

### 2.1 Replication of T2D subtypes in the extended QBB cohort

In this study of the extended QBB cohort, which included 13,808 participants, 2,765 were identified with T2D (20%) and 71 with T1D (0.5%). Subjects with T1D were removed, and clustering was performed on T2D subjects. The clinical measurement of T2D subtypes is shown in Table 1.

**Table 1:** Comparison of clinical characteristics of type 2 diabetes subtypes in the QBB cohort.

Cohort	SIDD	SIRD	MOD	MARD	p-value
Sample size (n)	495	116	1080	996	–
Sex (male%)	242 (48.8%)	53 (45.6%)	393 (36.38%)	436 (43.7%)	–
Age (years)	51.3 ± 10.17	51.5 ± 13.8	58.5 ± 8.66	44.6 ± 11.06	<2.2 × 10^−16^
Age of diagnosis (years)	39.9 ± 11.37	38.3 ± 13.05	54.9 ± 8.71	34.9 ± 8.54	<2.2 × 10^−16^
Physician diagnosis (n)	452 (91.3%)	101 (87.06%)	870 (80.5%)	901 (90.4%)	–
Diabetes treatment (n)	301 (60.8%)	75 (64.6%)	488 (45.1%)	436 (43.7%)	–
Glucose >200 mg/dL (n)	335 (76.6%)	31 (26.7%)	71 (6.5%)	36 (3.6%)	–
HbA1C > 6.5% (n)	495 (100%)	88 (75.8%)	765 (70.8%)	474 (47.5%)	–
BMI (kg/m^2^)	32.3 ± 6.1	34.04 ± 5.67	33.9 ± 6.2	30.12 ± 4.71	<2.2 × 10^−16^
LDL-C (mmol/L)	2.97 ± 0.99	2.62 ± 0.91	2.69 ± 0.98	2.77 ± 0.94	3.8 × 10^−5^
HDL-C (mmol/L)	1.21 ± 0.318	1.13 ± 0.28	1.31 ± 0.34	1.35 ± 0.39	<2.2 × 10^−16^
Total cholesterol (mmol/L)	5.11 ± 1.206	4.66 ± 1.033	4.74 ± 1.07	4.81 ± 1.03	2.5 × 10^−7^
Triglyceride (mmol/L)	2.101 ± 1.49	2.02 ± 1.08	1.6 ± 0.74	1.53 ± 0.903	<2.2 × 10^−16^
C-peptide (ng/ml)	2.51 ± 1.55	4.3 ± 3.83	3.05 ± 1.53	2.5 ± 1.31	<2.2 × 10^−16^
C-peptide fasting (ng/ml)	2.32 ± 1.17	2.606 ± 2.43	2.72 ± 1.27	2.32 ± 0.933	<2.2 × 10^−16^
Insulin (μU/ml)	17.4 ± 12.6	98.3 ± 70.9	16.5 ± 11.32	13.95 ± 9.66	<2.2 × 10^−16^
Fasting insulin (μU/ml)	15.12 ± 10.55	87.04 ± 49.27	14.79 ± 9.42	12.78 ± 8.21	<2.2 × 10^−16^
Glucose (mmol/L)	13.13 ± 3.75	8.99 ± 5.07	7.37 ± 2.207	6.56 ± 2.07	<2.2 × 10^−16^
Fasting glucose (mmol/L)	12.69 ± 3.62	7.82 ± 4.17	7.27 ± 1.95	6.56 ± 1.87	<2.2 × 10^−16^
HbA1C (%)	10.13 ± 1.48	7.85 ± 1.75	6.94 ± 0.93	6.403 ± 0.95	<2.2 × 10^−16^
HOMA2-%B	35.6 ± 28.16	312.13 ± 176.34	85.5 ± 48.12	95.05 ± 52.41	1.9 × 10^−6^
HOMA2-IR	3.07 ± 2.28	12.92 ± 7.74	2.31 ± 1.63	1.89 ± 1.33	<2.2 × 10^−16^
eGFR (mL/min/1.73m^2^)	102.5 ± 17.6	98.6 ± 23.7	96.02 ± 16.21	108.47 ± 14.84	<2.2 × 10^−16^
AST (U/L)	20.5 ± 14.1	20.8 ± 9.6	20.1 ± 8.9	18.8 ± 7.4	2.1 × 10^−7^
ALT (U/L)	27.8 ± 22.1	27.2 ± 16.4	23.9 ± 14.7	23.1 ± 13.9	1.3 × 10^−6^

The data indicate numbers (n) with percentages and mean ± standard deviation as appropriate. The p-values from Kruskal–Wallis rank sum test. ALT; Alanine aminotransferase. AST; Aspartate aminotransferase. HbA1C; Hemoglobin A1c. LDL-C; Low-density lipoprotein cholesterol. HDL-C: High-density lipoprotein cholesterol. eGFR; estimated Glomerular filtration rate. HOMA; Homeostasis model assessment estimation of beta cell function (%B) and insulin resistance (IR).

**Figure 1:**
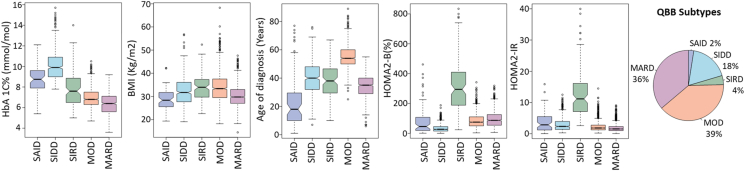
**Distribution of Type 2 diabetes subtypes in the QBB cohort derived by k-means clustering.** The figure illustrates the subtypes of diabetes among 2,687 clustered individuals with T2D. Subjects with T1D (SAID; n=68) were not included in the clustering but are shown for comparison. The x-axis on the box plots categorizes the subtypes, and the y-axis displays corresponding values of HbA1C, BMI, age of diagnosis, and HOMA2 levels (HOMA2-%B and HOMA2-IR). Non-overlapping of the notches, which represent the 95% confidence interval around the median, indicate statistically significant differences between the subtypes. The pie chart illustrates the rounded-off percentages of each subtype in the QBB cohort. QBB: Qatar Biobank; SAID: Severe autoimmune diabetes; SIDD: Severe insulin-deficient diabetes; SIRD: Severe insulin-resistant diabetes; MOD: Mild obesity-related diabetes; MARD: Mild age-related diabetes.

The clustering analysis included 2,687 T2D subjects, who had all the variables required for clustering. Using k-means clustering, we categorized all T2D participants into four subtypes (Figure 1). Consistent with previous studies, SIDD exhibited the highest HbA1C levels. Both MOD and SIRD had the highest BMI among the groups. Notably, MOD individuals were diagnosed at an older age compared to other subtypes, whereas SAID individuals (prevalance of 2.1%) had the earliest diagnosis. SIRD individuals demonstrated the highest values for insulin resistance (HOMA2-IR) and beta-cell function (HOMA2-%B). The distribution of T2D subtypes within the cohort showed that MOD was the most prevalent subtype at 39.4%, followed by MARD at 36.3%, SIDD at 18.0%, and SIRD at 4.2%.

### 2.2 Assessing the cluster reproducibility by applying coordinates from the ANDIS cohort

We used ANDIS cohort coordinates on QBB individuals to test our data-driven clusters' reproducibility (Fig. S1A). Two main differences emerged: while ANDIS coordinates showed MOD as the cluster with the lowest age of diagnosis among T2D clusters, QBB coordinates indicated MARD. However, regardless of the coordinate system used, MARD consistently exhibited significantly lower BMI, HbA1c, and HOMA2-IR values compared to other T2D subtypes (Fig. S2A–B). Furthermore, the most prevalent subtype shifted from MARD in the ANDIS-based classification to MOD in the QBB-based classification.

To visualize the shifts in cluster assignment within the QBB when employing ANDIS coordinates compared to original QBB clusters, a sankey plot was used (Fig. S3A). Notably, SIDD subtype demonstrated the most stability in cluster membership. However, with QBB coordinates, only 4% of individuals were categorized as SIRD, but this increased to 11% with ANDIS coordinates, as many initially classified as MOD under QBB were reassigned to SIRD using ANDIS. Over half of the initial clusters for MOD and MARD showed changes when using ANDIS coordinates. The significant shift underscores the advantages of employing population-specific derived cluster centers over using coordinates from disparate population studies.

### 2.3 Assessing the impact of using actual age versus age of Diagnosis in Clustering

To evaluate the impact of replacing the age of diagnosis with actual age on clustering, we analyzed a subset of 1,772 individuals (65% of our cohort) with complete data for five variables: HbA1C, Age of diagnosis, BMI, HOMA2-%B, and HOMA2-IR. Clustering was first performed using the recorded age of diagnosis, followed by a second analysis in which this variable was substituted with the participants’ actual age. The results revealed notable differences in clustering outcomes depending on the age metric used. When the age of diagnosis was applied, MOD was identified as the predominant subtype (Fig. S1B). In contrast, using actual age shifted the classification, making MARD the predominant subtype (Fig. S1C). Sankey plot illustrates the retention and changes in cluster assignments when switching from the age of diagnosis to the actual age (Fig. S3B). The analysis showed that SIDD and SIRD were more stable, maintaining consistent memberships, while MOD and MARD exhibited greater variability, retaining only ∼50% of their original clusters. This finding highlights the sensitivity of MOD and MARD classifications to the choice of age metric. The results emphasize the significant impact of using actual age on clustering outcomes for these subtypes, highlighting the importance of carefully selecting the age variable in diabetes subtype analysis.

### 2.4 Assessing the effect of diabetes duration on the cluster stability

We conducted additional analysis to evaluate the cluster stability based on the classification of all individuals with T2D (Fig. S4A) compared to those with a diabetes duration of less than five years (Fig. S4B). In the QBB cohort, there were 401 individuals with T2D for less than five years. The clusters SIDD, MOD, and MARD showed high stability, retaining their cluster membership at rates of 92%, 85%, and 98%, respectively (Fig. S4C). The SIRD cluster, which included 28 individuals, exhibited a retention rate of 36%. This is likely due to SIRD typically developing over an extended period, and restricting the analysis to 5 years only captures a small proportion of SIRD patients. Analyzing the time since diagnosis for the entire QBB cohort revealed that SIRD had the longest duration (13.2 years) compared to the other three subtypes (8.2 years).

Furthermore, Individuals categorized as SIRD within the less than five years diabetes duration group do not represent those with extreme HOMA2-IR values. Hence, cluster stability does not appear to be significantly influenced by the duration of diabetes, as SIDD, MOD, and MARD retained over 85–98% of their cluster membership.

### 2.5 Identifying sex differences in T2D subtypes within the QBB cohort

**Figure 2:**
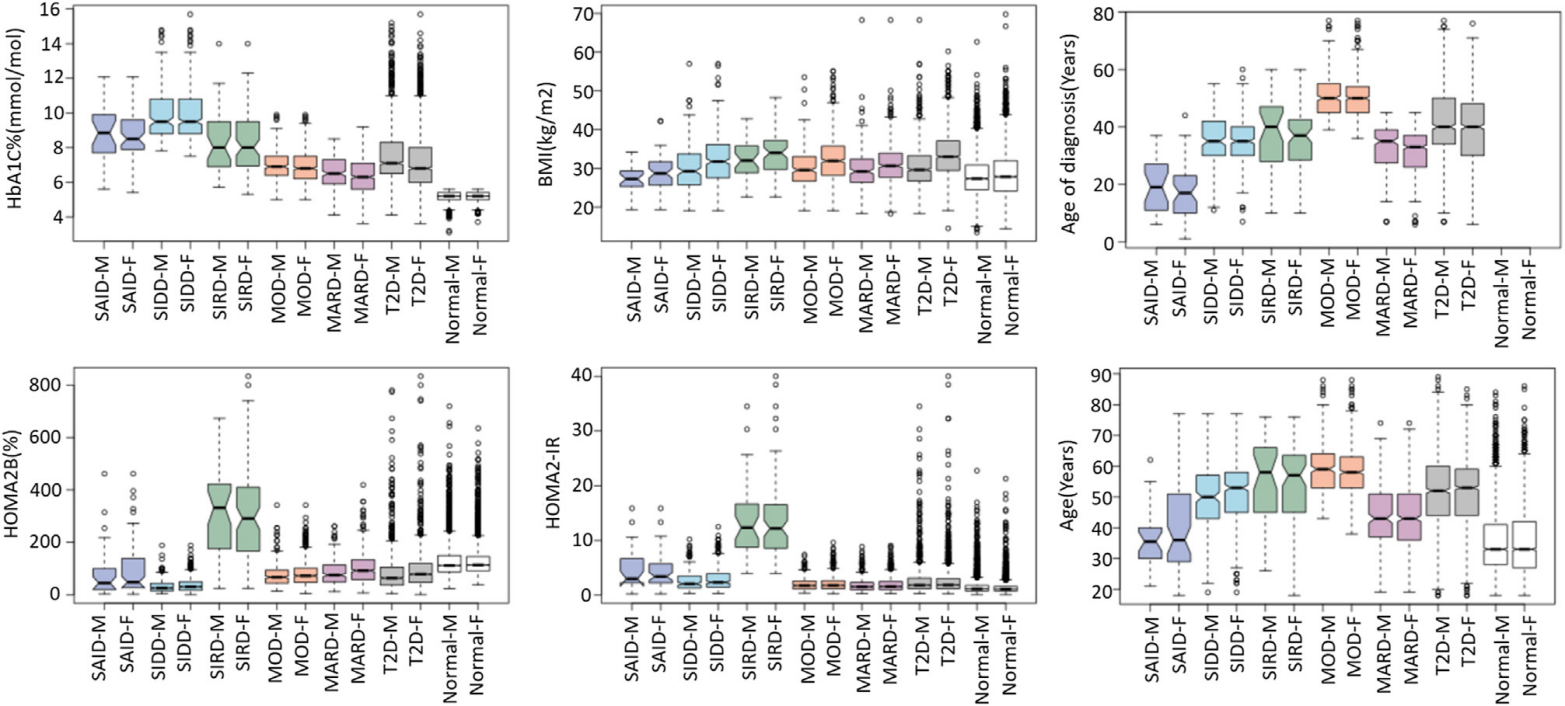
**Distribution of key cluster variables by sex across diabetes subtypes in the QBB cohort.** The distribution of HbA1C, BMI, age of diagnosis, age, and HOMA2 levels (HOMA2-%B and HOMA2-IR) across T2D subtypes, The x-axis on bar plots represent T2D subtypes (SIDD, SIRD, MOD, MARD), T1D (SAID) and normal subjects, categorized by sex (M: male, F: female), and the y-axis shows the corresponding values for each metric. Non-overlapping of the notches, which represent the 95% confidence interval around the median, indicates significant differences between the subtypes. T1D (SAID) and normal subjects were not included in the clustering but are shown for comparison.

To identify sex differences within the QBB cohort, we split the subtypes into males and females and examined variations in the five variables (HbA1C, BMI, Age of diagnosis, HOMA2-%B, and HOMA2-IR). We also compared the individuals having T2D with a control group of normal individuals to ensure the identified differences were unique to T2D (Figure 2 and Table S1). Significant sex disparities emerged predominantly in the MARD subtype, where males exhibited higher HbA1C levels than females. Across all subtypes (SIDD, SIRD, MOD, MARD), females consistently showed higher BMI values than their male counterparts. The analysis of age metrics revealed significant differences between males and females in MOD and MARD. MOD individuals were generally older at diagnosis compared to other subtypes. Specifically, the average age at diagnosis for MOD males was 52.8 years, increasing to 59.5 years when considering actual age. For MOD females, these figures were 50.5 and 57.8 years, respectively. In contrast, MARD males had an average age of diagnosis of 36.1 years, which rose to 46.1 years for actual age, while MARD females were diagnosed at an average age of 32.8 years and had an actual age of 43.4 years. In terms of beta-cell function, females exhibited significantly higher HOMA2-%B levels in MARD and SIDD subtypes compared to males, highlighting a notable sex difference within these subtypes. For HOMA2-IR levels, only SIDD showed a significant difference between the sexes (Table S1). This analysis underscores the importance of considering sex differences in the clinical evaluation and management of diabetes, as these variables influence disease presentation and progression significantly within different T2D subtypes.

### 2.6 Insulin treatment across T2D subtypes in the QBB cohort

In the QBB cohort, both SIDD and SIRD subtypes displayed significantly higher HbA1C levels compared to those with MOD and MARD. Consequently, it was anticipated that SIDD and SIRD would require more frequent insulin treatment to manage their elevated HbA1C levels. According to the responses from the QBB questionnaire, a high percentage of insulin prescriptions were indeed observed in these groups. Specifically, the distribution of insulin treatment was as follows: 41.4% for SIDD, 62.9% for SIRD, 12.8% for MOD, and 14.4% for MARD. This pattern underscores the need for targeted insulin therapies, particularly in subtypes where hyperglycemia is more pronounced.

### 2.7 Evaluating MODY subjects with T2D subtypes

**Figure 3:**
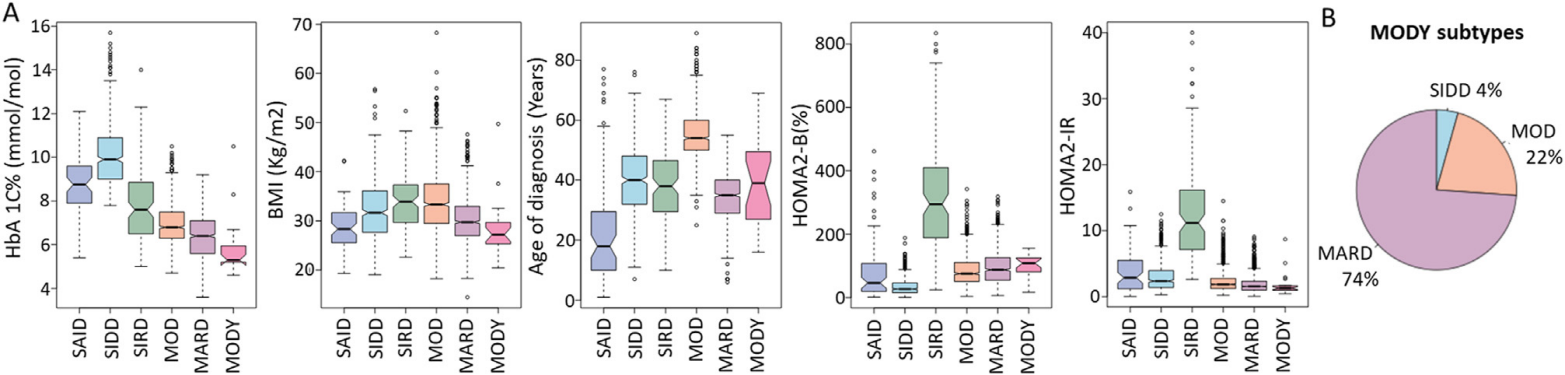
**Comparative analysis of MODY individuals and T2D subtypes in the QBB cohort. A.** An assessment was conducted on the 24 MODY individuals, evaluating them across the five variables (HbA1C, BMI, age of diagnosis, HOMA2-%B and HOMA2-IR). **B.** Pie chart represents the classification of MODY into different T2D subtypes. Non-overlapping of the notches, which represent the 95% confidence interval around the median, indicates significant differences between the subtypes. T1D (SAID) subjects were not included in the clustering but are shown for comparison.

Our previous study [13] identified 24 individuals who were initially misclassified as having T2D but were later confirmed to have maturity-onset diabetes of the young (MODY) through genetic analysis. These 24 individuals carried mutations annotated as pathogenic and likely pathogenic MODY-causing mutations in the Human Gene Mutation Database (HGMD). In our current analysis, we extracted these individuals and analyzed their data across five parameters: HbA1C, BMI, Age of diagnosis, HOMA2-%B, and HOMA2-IR as shown in Figure 3A. The analysis revealed that MODY individuals typically exhibit lower levels of HbA1C, BMI, and HOMA2-IR, which more closely resemble the characteristics of MARD subtype. Furthermore, when clustering MODY among T2D individuals, the majority of MODY cases fell under the MARD category, with 17 out of 24 individuals (approximately 74%) falling into this subtype. A smaller number of individuals were classified as MOD (n = 5; 22%), and only one was categorized as SIDD as depicted in Figure 3B. This clustering approach helps delineate the distinct metabolic profiles of MODY within the broader context of T2D subtypes.

### 2.8 Assessment of chronic kidney disease and metabolic dysfunction-associated steatotic liver disease (MASLD) prevalence in the QBB cohort

**Figure 4:**
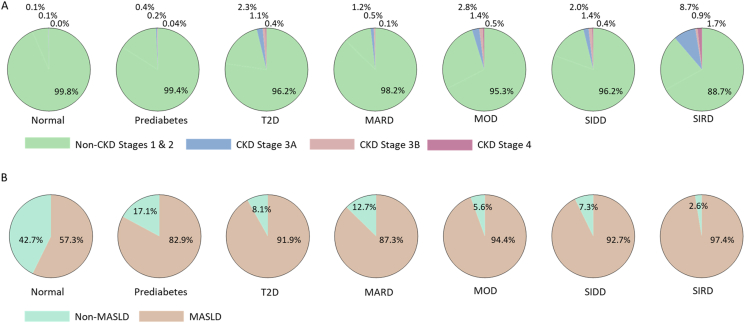
**The Prevalence of CKD and MASLD in QBB cohort across different categories and T2D subtypes.A.**Distribution of kidney function among individuals categorized as Normal, Prediabetes, and all T2D, followed by the distribution of kidney function among T2D subtypes (MARD, MOD, SIDD, and SIRD). **B.** Distribution of MASLD among individuals categorized as Normal, Prediabetes, and all T2D, followed by the distribution of MASLD among T2D subtypes (MARD, MOD, SIDD, and SIRD). Percentage for CKD prevalence and MASLD listed for each group.

We analyzed the prevalence and stage of CKD across the QBB cohort. CKD was categorized into five stages based on estimated Glomerular Filtration Rate (eGFR): stage 1 indicating normal kidney function (eGFR >90 ml/min/1.73 m^2^), stage 2 for mild loss (eGFR 89 to 60 ml/min/1.73 m^2^), stage 3A for moderate loss (eGFR 59 to 45 ml/min/1.73 m^2^), stage 3B for more significant moderate loss (eGFR 44 to 30 ml/min/1.73 m^2^), and stage 4 for severe loss (eGFR 29 to 15 ml/min/1.73 m^2^). The analysis revealed that a significant number of T2D individuals progressed to various stages of CKD (Figure 4A), with most CKD severe stages falling in T2D cases (stage 3A: 2.3%; stage 3B: 1.1%; stage 4: 0.4%). In contrast, individuals without diabetes (Normal) predominantly remained within the normal kidney function, and only a few had mild to moderate loss (stage 3A: 0.1%; stage 3B: 0.1%). Pre-diabetic individuals primarily exhibited normal kidney function, with a minority progressing to more severe CKD (stage 3A: 0.4%; stage 3B: 0.2%; stage 4: 0.04%). This finding underscores that CKD occurrences are notably higher among individuals with T2D compared to those without diabetes. Further stratification assessed CKD risks within each T2D subtype. The SIRD subtype showed the highest prevalence of CKD (stage 3A: 8.7%; stage 3B: 0.9%; stage 4: 1.7%), whereas a smaller proportion of those with SIDD or MOD were affected (SIDD, stage 3A: 2%; stage 3B: 1.4%; stage 4: 0.4%; MOD, stage 3A: 2.8%; stage 3B: 1.4%; stage 4: 0.5%). The MARD group displayed the lowest prevalence of CKD (stage 3A: 1.2%; stage 3B: 0.5%; stage 4: 0.1%). These findings provide insights into the differential risks of kidney disease across diabetes subtypes, indicating a need for targeted clinical monitoring and intervention strategies.

Next, we determined the prevalence of MASLD in the QBB cohort and investigated the prevalence among the different diabetes subtypes. MASLD was identified based on the evaluation of the hepatic steatosis index (HSI) and fulfillment of diagnostic clinical criteria for MASLD [16]. HSI is widely used as a robust screening tool for MASLD (NAFLD). Results showed high MASLD prevalence among normal, prediabetes, and T2D individuals, while T2D showed the highest MASLD prevalence (Figure 4B). Stratification of MASLD within diabetes subtypes showed the highest prevalence in SIRD (97.4%), followed by MOD (94.4%), SIDD (92.7%), and MARD (87.3%). Combined, the QBB cohort showed high MASLD prevalence evident from HSI distribution. Interestingly, the distribution between T2D subtypes showed the lowest prevalence in MARD.

We checked for significant differences in the occurrence of CKD among various T2D subtype pairs using Fisher's exact test (Table S2). Individuals with kidney function stages 1 or 2 were considered healthy, while those in stages 3A, 3B, or 4 were considered to have CKD. The analysis revealed that SIRD subtype had a significantly higher prevalence of CKD compared to SIDD, MOD, and MARD. SIDD had a higher prevalence compared to MARD, while differences between MOD and SIDD in CKD were not statistically significant. Lastly, MOD had a higher prevalence compared to MARD. Comparing the prevalence of CKD for T2D subtypes compared to healthy normal showed a similar trend of CKD prevalence in which SIRD had the highest prevalence of CKD, followed by MOD, then SIDD, and least on MARD, compared to the healthy normal group (Table S3). CKD prevalence in QBB based on ANDIS coordinates showed that the prevalence of CKD was significantly higher for SIRD only compared to the other subtypes (Table S4). Meanwhile, SIDD, MOD, and MARD had no significant differences in the prevalence of CKD. Therefore, using QBB coordinates resulted in a better assessment of CKD prevalence among the subtypes than using ANDIS coordinates. We checked for significant differences in the prevalence of MASLD between T2D subtypes, which showed that SIRD, SIDD, and MOD individuals had significantly higher MASLD prevalence compared to MARD (Table S5), however, there were no statistically significant differences between MASLD prevalence among other subtypes (SIDD, SIRD, and MOD).

We further assessed CKD risk in MODY individuals, as detailed in Table S6. Since the majority of MODY individuals clustered into MARD, we would expect MODY individuals to have a lower risk of CKD. The findings highlighted that the majority of MODY clustered into MARD maintained normal kidney function (stage 1), in contrast to those in SIDD (stage 3B) or MOD (stages 1, 2, or 4), which had a higher prevalence of CKD.

### 2.9 Model prediction of diabetes subtypes and diabetes complications in the QBB cohort

We assessed two models: one predicting diabetes subtypes and the other predicting complications. Using clustering parameters, we split the data into 75% for training and 25% for testing, and used 10-fold cross validation (CV). The diabetes subtype prediction model achieved a 95% accuracy with GLM (95% CI: 0.93–0.97), Sensitivity 99%, Specificity 99%, P-value <2.2 × 10^−16^, validated by 10-fold CV. For predicting diabetes complications, we evaluated CKD and MASLD. For predicting CKD, the model did not perform well (50% accuracy); due to the limited number of CKD samples in the testing cohort. However, the model achieved good results for MASLD with 92% accuracy [95% CI: 0.90–0.94], Sensitivity 96%; Specificity 63%; and P-value 5.8 × 10^−5^ with 10-fold CV.

## 3 Discussion

In this study, we were able to replicate [4] T2D subtypes (SIDD, SIRD, MOD, and MARD), not including the other subtypes identified in other populations [11]. In the QBB cohort, although MARD individuals were younger than other subtypes, they exhibited significantly lower BMI, HbA1C, and HOMA2IR compared to other T2D subtypes, in agreement with the classification proposed by Ahlqvist et al., indicating a milder form of T2D. The analysis shows that data-derived centers outperform ANDIS coordinates for classifying T2D in QBB. We expect errors in the reported age of diagnosis mainly influenced the classification of MOD and MARD, but did not impact SIDD and SIRD. Diabetes duration did not affect the cluster assignment of SIDD, MOD, and MARD clusters in the QBB. Despite significantly higher BMIs in females compared to males within the QBB, this did not translate to increased disease severity, as indicated by comparable HbA1C, HOMA2-%B, and HOMA2-IR levels across sexes. We recommend employing clustering methods in populations lacking genetic testing to better identify misclassified MODY individuals within the MARD group. Additionally, CKD prevalence was highest in SIRD, followed by MOD and SIDD, and was lowest in MARD. Similarly, MASLD was predominantly prevalent in SIRD, followed by MOD and SIDD, and lowest in MARD. When the data were split into training and testing sets, we were able to predict the subtypes well in the testing set. As for the model prediction of diabetes complications, the model had a good performance only for MASLD but not for CKD, which is attributed to the small number of individuals with CKD within the Cohort.

### 3.1 Population-specific features of QBB T2D subtypes

In the QBB cohort, the MOD subtype exhibited significantly lower HbA1C levels compared to SIRD subtype, a finding that diverges from results observed in other populations [4,19]. This divergence may be attributed to genetic, environmental, or lifestyle factors unique to the Middle Eastern population. For instance, the high prevalence of consanguinity in the Qatari population could contribute to the observed differences. Previous studies [20] have noted a frequent family history of diabetes in MOD but not in MARD. However, a high prevalence of diabetes family history was reported across all four subtypes in the QBB - 87% in SIDD, 88% in SIRD, 81% in MOD, and 90% in MARD, which may be attributed to the high rates of consanguinity in the Qatari population [21].

Several studies [4,15,[22], [23], [24]] have shown that MARD typically presents with a later age of diagnosis, yet this trend was not observed in the QBB cohort (Fig. 1). The age of diagnosis for the QBB cohort and the Korean population [15] was lower compared to the ANDIS cohort [4]. Additionally, in the QBB cohort, MOD individuals were older than MARD individuals (Figure 2), suggesting that in the QBB, those classified as MOD are older T2D individuals diagnosed at a later age than those in MARD. This finding is unique to the QBB population and has not been documented before.

Since MOD and MARD are primarily distinguished by their BMI levels: high for MOD and low for MARD, we further analyzed beta cell function using HOMA2-%B. We found that MARD exhibited significantly higher HOMA2-%B compared to MOD (Figure 1, Figure 2). However, when using ANDIS coordinates, MOD showed higher HOMA2-%B compared to MARD (Fig. S1A), which may be attributed to the reclassification of some individuals from MARD to MOD (Fig. S3A). In a study of the Indian population, utilizing ANDIS coordinates led to a higher classification of individuals into MOD and fewer into SIDD and MARD, in contrast to classifications using data-derived centroids [25]. Therefore, using data-derived centers is recommended for more accurate T2D classification.

Age versus age at diagnosis in clustering resulted in classifying T2D into four subtypes, in which the SIDD group had an extreme level of HbA1C, the SIRD group had an extreme level of HOMA2-IR, while the MOD group had an extreme BMI level, and MARD had lower levels for these three variables. We found that the classification using age versus age at diagnosis does affect the majority of the classification of MOD and MARD clusters (Fig. S3B). However, the majority of SIRD clusters remained unchanged, and SIDD had only a mild change in cluster assignment. Further research is needed to explore these factors and their implications for treatment strategies.

### 3.2 Phenotypic differences between QBB T2D males and females

The majority of T2D males in the QBB cohort had significantly lower BMI than T2D females (Figure 2; Table S1). Although females had significantly higher BMI, they did not have significantly higher HbA1C levels or HOMA2-IR compared to males. The prevalence of obesity is more common in females than males globally [26]. Another notable sex difference is observed in the MARD subtype: females had significantly lower HbA1C levels compared to males, while the opposite was true for their HOMA2-%B levels (Figure 2; Table S1). This indicates that MARD females had better beta-cell function, enabling them to maintain better control of their HbA1C levels compared to MARD males (Figure 2).

A similar sex difference in BMI was observed in the Emirati population, where T2D females were more obese than males as well [27]. However, to the best of our knowledge, this is the first report of a significant BMI difference across the four subtypes by sex, highlighting the potential need for different personalized treatments based on sex.

### 3.3 The prevalence of chronic kidney disease (CKD) in T2D subtypes

An increased risk of renal and cardiovascular events is typically observed in SIDD, SIRD, and MOD subtypes, but not in MARD [14]. Contrarily, in the Korean population, higher CKD events were noted in the SIRD, SIDD, and MARD subtypes, with MOD showing lower prevalence [15]. The SIDD and SIRD subtypes had higher events of CVD compared to MOD [28]. Additionally, retinopathy and neuropathy were more prevalent in SIDD, whereas diabetic kidney and fatty liver disease affected individuals in the SIRD group [4].

In the QBB cohort, due to limited data on cardiovascular risk, our analysis primarily focused on assessing the risk of CKD events (Figure 4A). CKD was more prevalent among T2D individuals compared to the other categories (Normal and Prediabetes). Further analysis of CKD prevalence by subtype revealed the highest rates in SIRD individuals, followed by SIDD and MOD (Table S2; S3). These findings are consistent with previous reports [4,14] yet contrast with data from the Korean population, which showed a higher risk of CKD in MARD than MOD [15].

### 3.4 The prevalence of metabolic dysfunction-associated steatotic liver disease (MASLD) in T2D subtypes

MASLD diagnosis involves evaluation of hepatic steatosis in the presence of metabolic dysregulation and one of five cardiometabolic risk factors, which include BMI ≥25 kg/m^2^, fasting serum glucose ≥5.6 mmol/L, HbA1c ≥ 5.7%, or T2D [16]. The QBB cohort presented with a high prevalence of these risk factors (Figure 4B). Non-invasive tools are widely utilized for effectively evaluating MASLD, and HSI has previously shown comparable performance to other indicators of hepatic steatosis, such as Fatty Liver Index (FLI), triglyceride, and glucose (TyG) index [29]. HSI calculation combined with diagnostic criteria for MASLD indicated high MASLD prevalence in our study cohort. Notably, our assessment of MASLD relied on HSI calculation that includes BMI in its derivation, and the presence of an additional comorbidity, which also included BMI. Around 70% of the Qatari population has been previously reported to be overweight or obese (BMI>25) [30]. The high MASLD prevalence (∼57% of the non-diabetes subjects) reported herein could be driven by these factors, reflecting a seemingly high overestimation compared to recent predicted estimates of MASLD prevalence in Qatar at ∼44% in the general population and ∼77% among T2D [31]. MASLD is a common comorbidity among T2D individuals, but the highest prevalence was recorded in SIRD (Figure 4B; Table S5). These findings are supported by the German Diabetes Study, which reported the highest NAFLD fibrosis scores, fatty liver index, and AST/platelet ratio in SIRD individuals [9]. Moreover, SIRD individuals also previously showed higher liver fibrosis (Fibrosis-4: FIB-4) scores than SIDD and MOD individuals [17]. Overall, the distribution of hepatic steatosis patterns within diabetes subtypes presented herein reflects the applicability of HSI for predicting MASLD in individuals who may require timely interventions for mitigating the risk of advanced liver diseases such as Metabolic Dysfunction-Associated Steatohepatitis (MASH) and cirrhosis. The higher prevalence of CKD and MASLD in certain subtypes warrants a more detailed analysis of the potential mechanisms driving these risks.

### 3.5 Potential treatment approaches for T2D subtypes

Among the T2D subtypes, SIDD and SIRD exhibit severe disease progression. Studies [32] have shown that individuals with less than eight years of T2D duration who underwent Laparoscopic Sleeve Gastrectomy (LSG) achieved complete diabetes remission along with significant reductions in weight, dyslipidemia, and hypertension. Furthermore, Raverdy et al. [33] reported that SIRD individuals experienced substantial improvement in renal function and higher rates of diabetes remission following bariatric surgery. In terms of pharmacological treatment, SIRD individuals have shown positive responses to thiazolidinedione therapy, which effectively lowers HbA1C levels [34]. Additionally, treatments such as pioglitazone, SGLT2 inhibitors (SGLT2i), and GLP-1 receptor agonists (GLP-1RA) have proven effective in halting the progression of NAFLD in SIRD [35].

Recent studies have shown that GLP1-RA treatment results in a stronger glycemic response in females than in males, according to both observational and clinical trials [36]. This indicates that females may experience more favorable responses to GLP1-RA than their male counterparts. However, additional research is necessary to explore these effects specifically at the subtype levels, rather than for T2D as a whole. Moreover, it is crucial to conduct further studies to monitor inflammatory markers in SIRD individuals, who have shown elevated levels of IL-6, EN-RAGE, and CASP-8 in this group [37]. Investigating potential differences in these inflammatory markers between sexes could yield a deeper understanding of the optimal treatment and management for SIRD.

In the QBB cohort, MARD individuals displayed the lowest prevalence of CKD (Figure 4A; Table S2; S3). These individuals also had lower HbA1C compared to other subtypes (Figure 1, Figure 2) and demonstrated the highest beta-cell function (HOMA2-%B). Research has indicated that the MARD subtype experiences a reduction in fasting glucose following metformin treatment [38]. Consequently, MARD individuals in the QBB may particularly benefit from metformin, a first-line medication for diabetes.

Insulin treatment is commonly prescribed for SIDD (41%) and SIRD (62%) individuals in the QBB cohort. Other T2D medications have been shown to reduce both mortality and cardiovascular events, presenting them as better alternatives to insulin [39]. Moreover, T2D individuals with high insulin resistance who are treated with insulin have a higher risk of diabetic kidney disease and cardiovascular events [40]. Additionally, those taking both insulin and other antidiabetic drugs exhibit a higher cardiovascular risk ratio compared to those on antidiabetic drugs alone [41].

### 3.6 Clustering of MODY individuals within the T2D framework

Our analysis showed that the majority of the 24 MODY individuals in the QBB [13] predominantly clustered into MARD (Figure 3B). We hypothesize that MODY patients, likely misclassified within the clustering scheme, tend to be grouped into MARD. It has been observed that MARD subtype responds well to sulfonylurea therapy [34]. Additionally, several studies have reported that individuals with *HNF1A* and *HNF4A* mutations show high sensitivity to sulfonylurea treatment [42,43]. In the QBB cohort, about 45% of the 24 MODY cases had these mutations [13]; however, at the time of data collection, these individuals were misdiagnosed as T2D and were on standard T2D medication (metformin) and did not receive proper treatment plans designated for individuals with *HNF1A* and *HNF4A* mutations. The tendency for MODY individuals to cluster into MARD could help explain why this subtype derives significant benefits from sulfonylurea therapy in the previous findings [34], as these individuals may be misclassified MODY patients rather than typical T2D, as in the QBB cohort. Therefore, enhancing access to genetic testing is essential in areas with high type 2 diabetes rates [44]. In populations lacking genetic testing, identifying MODY individuals among T2D patients who cluster into the MARD subtype and respond well to sulfonylurea therapy might be feasible.

In the QBB cohort, while some MODY individuals developed CKD, the majority retained normal kidney function (Table S6). Recent research, including a study on the Indian population [45], has noted a significant occurrence of nephropathy and retinopathy in MODY individuals. This may explain the CKD risk observed among MODY individuals in the QBB cohort (Table S6). However, a more comprehensive understanding of the CKD risk requires additional research, especially with a larger cohort of MODY individuals.

### 3.7 Study limitations

Our study has certain limitations, including the absence of risk assessments for CVD for the subtypes and the lack of GAD antibody measurements to confirm SAID individuals. To improve the model's accuracy, increasing the sample size and incorporating additional variables such as genetic markers and lifestyle factors could be beneficial.

## 4 Conclusion

In our comprehensive study of the Qatari population within the Qatar Biobank, we successfully classified 2,687 individuals with T2D into four distinct subtypes. Our findings illuminate several unique characteristics of these subtypes, thereby enriching the framework for personalized diabetes management in this Middle Eastern population. Notably, our study underscored significant sex-based differences in diabetes expression. Females, across all subtypes, exhibited higher BMI levels than their male counterparts, while MARD females demonstrated higher beta-cell function and lower HbA1C levels compared to MARD males. These sex-specific differences emphasize the need for tailored diabetes treatment strategies that consider both biological and sex-specific factors.

The clustering approach also revealed that many MODY individuals aligned with the MARD subtype, suggesting that misclassified MODY cases within MARD may account for the observed sensitivity to sulfonylurea therapy. This finding highlights the potential role of genetic testing in accurately identifying MODY cases within the T2D framework, which could improve treatment efficacy.

Our CKD analysis showed that SIRD, SIDD, and MOD subtypes face higher CKD and MASLD risks compared to MARD, with SIRD exhibiting the highest prevalence and most severe disease complications. These findings support the need for intensive monitoring and tailored therapeutic strategies, particularly for individuals at a high risk of disease complications. Overall, this study supports the use of T2D subtyping to advance personalized treatment strategies, contributing valuable insights to enhance diabetes management in Middle Eastern populations. By refining subtype identification and considering population-specific factors to improve patient outcomes and quality of life.

## 5 Methods

### 5.1 Study cohort

The QBB cohort is a population-based study in Qatar that recruited Qatari and long-term residents (≥ 15 years living in Qatar) aged 18 years and older [46]. Extensive baseline socio-demographic data, clinical and behavioral phenotypic data, and serum concentrations of HbA1c, triglycerides, glucose, C-peptide, creatinine, total cholesterol, LDL-C, and HDL-C, and multiple other clinical biochemistry parameters [47] have been measured at the central laboratory of Hamad Medical Corporation (HMC), Doha, Qatar, accredited by the College of American Pathologists. All participants provided informed consent, and the study received approval from the QBB Institutional Review Board (Protocol no. E-2019-QF-QBB-RES-ACC-0179-0104). At the time of this study, QBB data were available for 13,808 Qatari participants. T2D classification was based on self-reported physician diagnosis, use of diabetic medications, HbA1C level >6.5%, or random glucose levels >200 mg/dL. Individuals with T1D (SAID n = 71) were identified based on self-reported T1D, or C-peptide levels <0.5 nmol/L and exclusively receiving insulin treatment [12], as glutamic acid decarboxylase (GAD) antibodies measurements were not available in QBB.

### 5.2 Definition of T2D and controls

Subjects were defined as controls if all the following four conditions were satisfied: first, no self-reported physician diagnosis of diabetes; second, no self-reported treatment with any diabetes-specific medication; third, HbA1c < 5.7%; and fourth, random glucose level <200 mg/dL. T2D was defined if any one of the following four conditions was satisfied: first, having a physician diagnosis of diabetes based on the questionnaire (86% of all participants), second, being treated for diabetes based on the QBB questionnaire (62%), third, having an HbA1c > 6.5% (63%), or fourth, having random glucose >200 mg/dL (17%). Based on this definition, ∼20% of individuals were defined as having T2D. Individuals with HbA1C between 5.7% and 6.4% (n = 1845) were excluded.

### 5.3 Cluster analysis

K-means clustering was performed for all T2D individuals (including males and females) using HbA1C, BMI, age of diagnosis, HOMA2-%B, and HOMA2-IR, setting the number of clusters (k) to 4 to derive the four subtypes of T2D. For individuals missing the age of diagnosis (35%), their actual age was used as a substitute. HOMA2-%B and HOMA2-IR were calculated for all T2D individuals based on C-peptide levels using the HOMA2 calculator (University of Oxford, Oxford, UK) [48]. Patients defined as SAID were excluded from the clustering and assigned to their own subtype, and clustering was carried out on the patients with T2D only. Clustering analysis was carried out on standardized values, centered at a mean of 0 and a standard deviation of 1 for all T2D individuals, combined males and females. The optimal number of clusters was determined using the k-means function in the “cluster” and “factoextra” libraries in R (version 4.3.1). We determined the optimal number of clusters to be k = 4. QBB coordinates for T2D subtypes are listed in Table S7. This finding was consistent with that observed by Ahlqvist et al. The cluster variables in QBB followed a similar trend to the ANDIS cohort. We, therefore, assigned cluster labels based on the five clinical variable averages that were characteristic of each T2D subtype following Ahlqvist et al. [4].

### 5.4 Sensitivity analyses

We conducted several sensitivity analyses to assess the robustness of our clustering results. We evaluated the impact of using the ANDIS coordinates [4] compared to our data-derived coordinates for identifying T2D subtypes in the QBB cohort. Changes in cluster assignments were visualized using Sankey plots to track the flow of cluster membership between QBB and ANDIS coordinates. The optimal number of clusters was determined from the training set using the Mclust function in the “mclust” R library v.5.4.5. Additionally, we determined the optimal number of clusters using the elbow method (Fig. S5) and the Bayesian Information Criterion (BIC) for expectation-maximization, initialized by hierarchical clustering for parameterized Gaussian mixture models (Fig. S6). We computed the BIC for various cluster sizes (two to fifteen). Using k-means cluster analysis on standardized variables, we derived cluster center coordinates for different values of k. Using a voting scheme [49], we determined the optimal number of clusters to be k = 4. This finding was consistent with that observed by Ahlqvist et al. Cluster stability was assessed by Jaccard similarity [50] using 100 re-runs of the k-means procedure. The Jaccard similarity to the original clusters was 0.93, which is generally considered an acceptable threshold for cluster stability [50]. The cluster variables in QBB followed a similar trend to the ANDIS cohort. We, therefore, assigned cluster labels based on the five clinical variable averages that were characteristic of each T2D subtype following Ahlqvist et al. [4]. We examined the effect of different age variables on the clustering outcomes by performing clustering based on the reported age of diagnosis and then repeating the analysis using the actual age of the participants. Both analyses employed the same clustering parameters that were used in the initial analysis. We also evaluated the effect of diabetes duration on cluster stability during the initial duration of diabetes. A sensitivity analysis was conducted to assess the cluster change based on the classification done for all T2D individuals versus those with a diabetes duration of less than 5 years. We investigated potential sex differences among the T2D subtypes by splitting the subtypes dataset into male and female groups. We compared five variables (HbA1C, BMI, Age of diagnosis, HOMA2-%B, HOMA2-IR) using Mann–Whitney U test for data deviating from normal distribution, assessed by Shapiro–Wilk Test.

### 5.5 Analysis of insulin use, MODY classification, CKD, and MASLD risk across QBB subtypes

First, we used data from the QBB questionnaire to identify individuals prescribed insulin treatment, enabling a comparison across the subgroups. Next, we compared 24 MODY individuals identified in our previous study [13]. These subjects were identified as MODY by whole genome sequencing and carriage of known pathogenic and likely pathogenic MODY-causing mutations in the Human Gene Mutation Database (HGMD) in genes known to cause MODY (MODY1 to MODY14) [13]. These 24 MODY individuals were compared with T2D individuals using five variables (HbA1C, BMI, age of diagnosis, HOMA2-%B, and HOMA2-IR). These MODY individuals were then clustered alongside T2D individuals to stratify into T2D subtypes. Additionally, the prevalence of chronic kidney disease (CKD) was assessed by calculating the estimated glomerular filtration rate (eGFR) based on creatinine levels, sex, and age [51,52]. HSI was calculated using serum alanine aminotransferase (ALT) to serum aspartate aminotransferase (AST) ratios, BMI, diabetes status, and sex stratification, while the recommended HSI score threshold of 36 was used to indicate NAFLD (MASLD) presence [53]. MASLD diagnosis was based on the presence of additional clinical criteria indicating metabolic dysfunction and presence of at least 1 out of 5 cardiometabolic risk factors, as described previously [16]. Fisher's exact test was applied to identify which T2D subtype had a higher risk of CKD and MASLD.

We performed predictive models using the clustering parameters (HbA1C, Age of diagnosis, BMI, HOMA2-%B, and HOMA2-IR) and we split the data into training (75%) and testing (25%) sets with 10-fold corss validation. We evaluated two models, one to predict T2D subtypes (Model_T2DClusters) and one for each tested complication (T2DComplication); CKD or MASLD as follows:

Model_T2DClusters = (T2D_subtypes ∼ Age + HbA1C + BMI + HOMA2B + HOMA2IR)

Model_T2DComplication = (MASLD or CKD ∼ Age + HbA1C + BMI + HOMA2B + HOMA2IR)

## CRediT authorship contribution statement

**Nayra M. Al-Thani:** Formal analysis, Data curation, Writing – original draft, Investigation. **Shaza B. Zaghlool:** Writing – review & editing, Investigation, Formal analysis. **Salman M. Toor:** Investigation, Writing – review & editing. **Abdul Badi Abou-Samra:** Resources, Investigation. **Karsten Suhre:** Writing – review & editing, Funding acquisition, Supervision, Conceptualization, Resources, Project administration, Investigation. **Omar M.E. Albagha:** Resources, Project administration, Investigation, Writing – review & editing, Funding acquisition, Supervision, Conceptualization.

## Institutional Review Board statement

The study was conducted in accordance with the Declaration of Helsinki, and approved by the Institutional Review Board of Qatar Biobank (Protocol no. E-2019-QF-QBB-RES-ACC-0179-0104), Doha, Qatar.

## Declaration of competing interest

All authors declare no competing interests.

## Data Availability

Access can be obtained upon request from: https://www.qphi.org.qa/research/how-to-apply.
