# Supplementary material for: Subtyping of type 2 diabetes from a large Middle Eastern biobank: Implications for precision medicine
Source: Mol Metab. 2025 Jun 23;99:102195. doi: 10.1016/j.molmet.2025.102195 (PMC12281152; doi:10.1016/j.molmet.2025.102195)
Supplement: Multimedia component 1 [file mmc1.pdf]

Supplementary material

List:

Figure S1: Clustering of QBB individuals under different settings
Figure S2: Pairwise comparison of MARD versus T2D subtypes.
Figure S3: Changes in cluster membership due to varying clustering metrics.
Figure S4: Clustering of QBB individuals based on the duration of diabetes.
Figure S5: Elbow method for determining optimal K for T2D clusters in the QBB Cohort.
Figure S6: The Gaussian finite mixture model to determine optimal K for T2D clusters in the QBB Cohort.
Table S1: Comparison of cluster variables between males and females across different T2D subtypes.
Table S2: Comparison of kidney function between different T2D subtypes.
Table S3: Comparison of kidney function between “Normal” and “T2D subtypes”.
Table S4: Comparison of kidney function between T2D subtypes based on ANDIS coordinates classification.
Table S5: Comparison of MASLD between different T2D subtypes.
Table S6: Comparison of kidney function between the 24 MODY individuals in QBB.
Table S7: QBB coordinates for T2D subtypes.

Supplementary Figures:

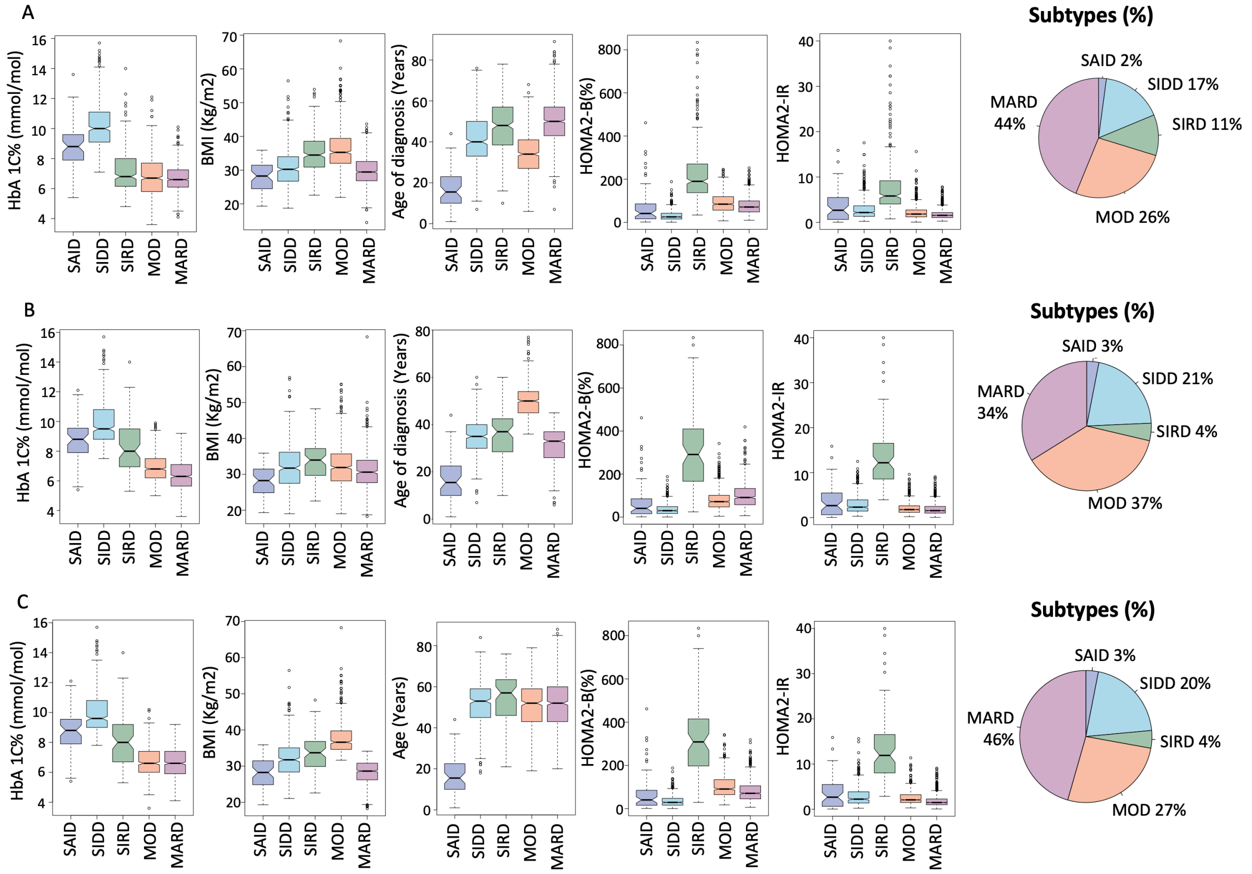

Figure S1: Clustering of QBB individuals under different settings. The x-axis lists subtypes, while the y-axis represents values of HbA1C, BMI, age of diagnosis, and HOMA2 levels (HOMA2-%B and HOMA2-IR). Subjects with T1D (SAID) were not included in the clustering but are shown for comparison. **A.** QBB subtypes were identified using ANDIS-derived coordinates. **B.** QBB subtypes were identified using the available age of diagnosis for 1,772 individuals and using QBB coordinates. The subtype features followed a similar distribution to the original clustering in QBB. **C.** QBB subtypes were identified using actual age instead of age of diagnosis for the same 1,772 individuals and using QBB coordinates.

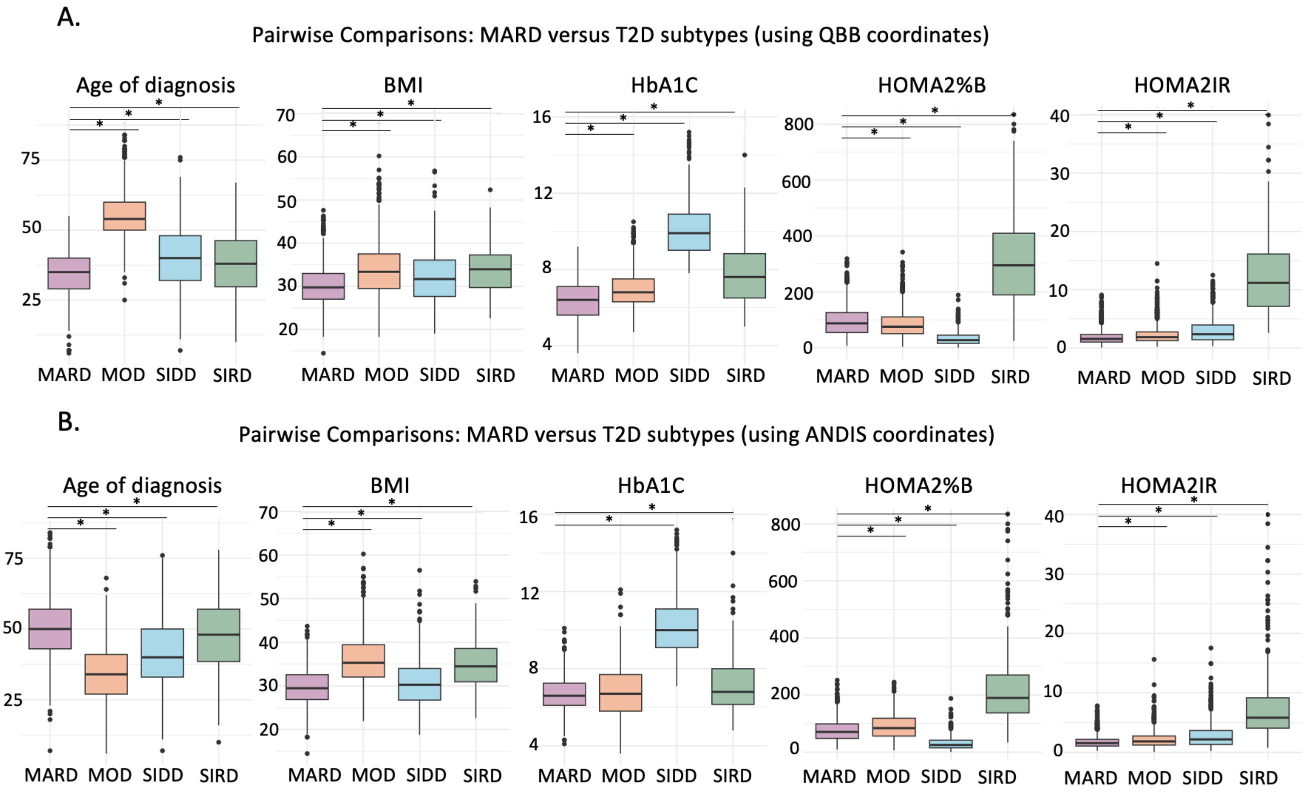

Figure S2: Pairwise comparison of MARD versus T2D subtypes using (**A**) QBB coordinates and (**B**) ANDIS coordinates. *P value < 0.05 from comparing MARD to other subtypes.

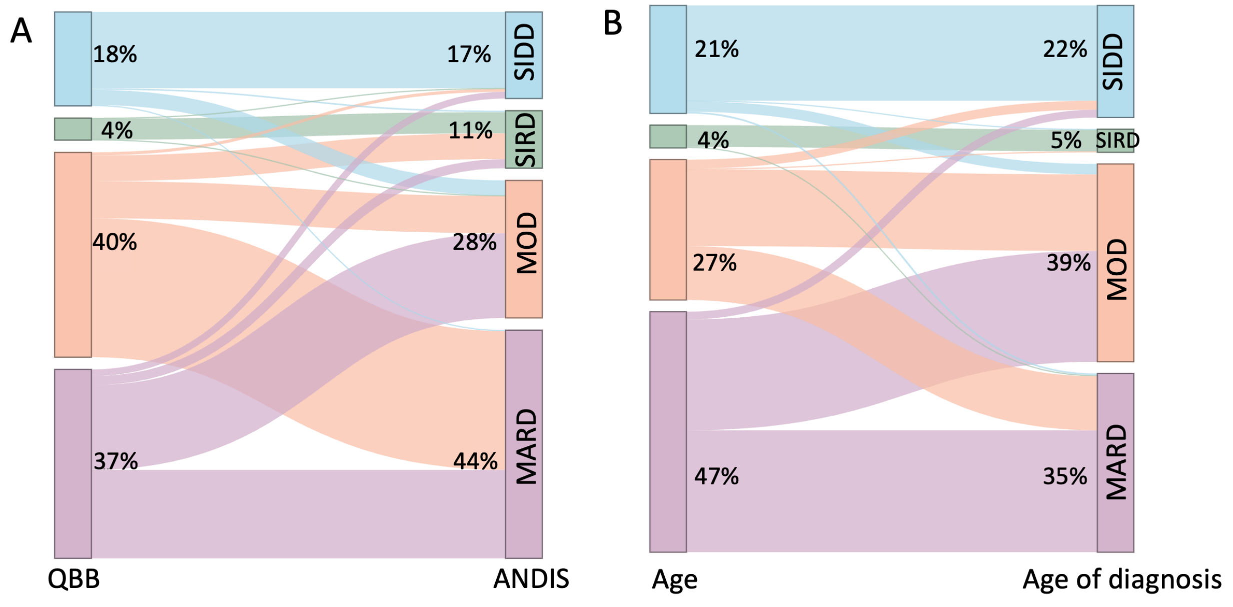

Figure S3: Changes in cluster membership due to varying clustering metrics. A. Cluster changes when using QBB-derived centers as opposed to using ANDIS coordinates. **B.** Cluster changes of T2D individuals when applying QBB coordinates and using the actual age instead of the age of diagnosis.

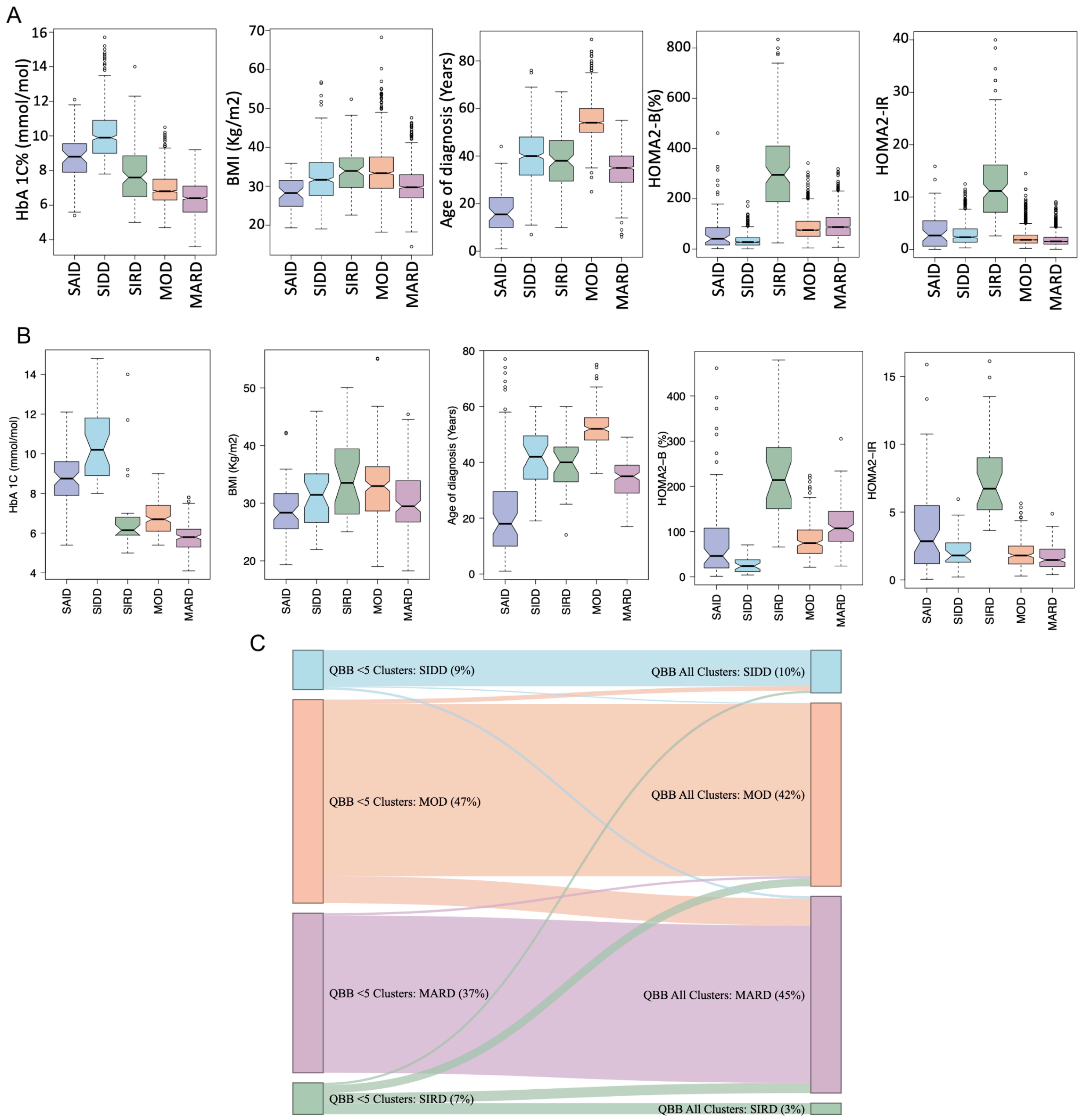

Figure S4: Clustering of QBB individuals based on the duration of diabetes. A. All individuals with T2D in the QBB cohort as depicted in Figure 1. **B.** Individuals with less than 5 years of diabetes duration. **C.** Sankey plot comparing T2D subtypes cluster assignment when considering all individuals versus only those with less than 5 years of diabetes duration (representing 15% of T2D within the QBB cohort).

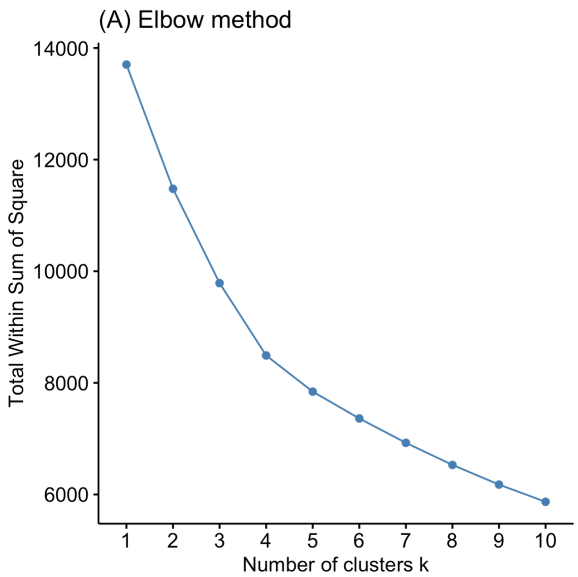

Figure S5: Elbow method for determining optimal K for T2D clusters in the QBB Cohort. The line plot represents the Elbow method for determining the optimal number of clusters in K-means. The x-axis shows the number of clusters (k) and the y-axis indicates the total within the sum of squares. The Elbow method shows the optimal number of K=4.

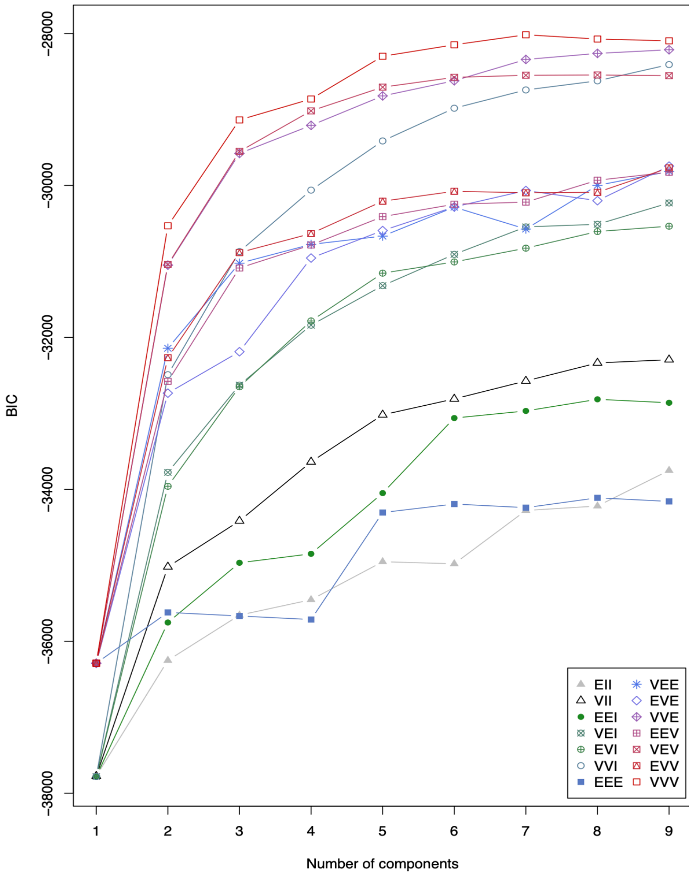

Figure S6: The Gaussian finite mixture model to determine optimal K for T2D clusters in the QBB Cohort. The Gaussian finite mixture model (GMM) shows the distribution of data combining multiple Gaussian distributions to determine subpopulations in the data and estimate the number of components. The x-axis shows the number of clusters (components). The y-axis shows the BIC (Bayesian Information Criterion) which indicates the value of each number of clusters (components). The best performing models based on different inclusion of covariance are VVV, VVE, VVI, and VEV.

Supplementary Tables:

Table S1: Comparison of cluster variables between males and females across different T2D subtypes.

T2D Subtypes		SIDD		SIRD		MOD		MARD	
**Variables**	**Sex**	Male	Female	Male	Female	Male	Female	Male	Female
**HbA1 C%**	**Mean (SD)**	10.1 ± 1.5	10.1 ± 1.4	7.8 ± 1.7	7.8 ± 1.7	7.1 ± 0.8	6.8 ± 0.9	6.6 ± 0.9	6.2 ± 0.9
	**p-value**	0.57		0.87		0.082		3.2×10^{-11}	
**BMI**	**Mean (SD)**	30.3 ± 5.6	34.2 ± 5.9	32.7 ± 5.4	35.1 ± 5.6	31.5 ± 5.8	35.3 ± 6.1	28.9 ± 4.3	31.1 ± 4.8
	**p-value**	7.1×10^{-14}		1.7×10^{-2}		2.2×10^{-16}		1.1×10^{-11}	
**Age of diagnosis**	**Mean (SD)**	37.2 ± 9.9	35.7 ± 9.3	38.1 ± 12.8	32.7 ± 9.3	52.8 ± 7.2	50.5 ± 6.7	36.1 ± 8.9	32.8 ± 8.1
	**p-value**	0.82		0.065		2.1×10^{-2}		1.3×10^{-8}	
**HOMA2-%B**	**Mean (SD)**	31.1 ± 26.1	38.9 ± 29.7	321.2 ± 174.8	304.4 ± 178.6	83.1 ± 48.8	86.8 ± 47.6	84.6 ± 51.3	103.1 ± 51.8
	**p-value**	4.9×10^{-3}		0.49		0.11		1.5×10^{-10}	
**HOMA2-IR**	**Mean (SD)**	2.8 ± 2.1	3.3 ± 2.3	13.7 ± 7.1	12.2 ± 8.2	2.3 ± 1.5	2.2 ± 1.6	1.9 ± 1.3	1.8 ± 1.3
	**p-value**	2.2×10^{-3}		0.082		0.27		0.95	

This table displays p-values calculated using Mann–Whitney U test to compare data points between males and females within each T2D subtype. It also describes the mean and standard deviation between males and females. Significant differences in HbA1C were observed only for MOD and MARD subtypes. Significant differences in BMI were observed between males and females across all four T2D subtypes. For Age of diagnosis, we observed significant differences for MOD and MARD subtypes. Significant differences for HOMA2-%B were observed only for SIDD and MARD subtypes. Significant differences in HOMA2-IR were observed only for SIRD subtype.

Table S2: Comparison of kidney function between different T2D subtypes.

Subtypes (% CKD)		*p*-value	Odds ratio (95% CI)
SIRD (11.3%)	SIDD (3.8%)	3.8×10^{-3}	3.17 (1.39 - 7.03)
SIRD (11.3%)	MOD (4.7%)	7.1×10^{-3}	2.56 (1.23 - 4.90)
SIRD (11.3%)	MARD (1.8%)	3.4×10^{-6}	6.91 (3.01 - 15.3)
SIDD (3.8%)	MARD (1.8%)	2.1×10^{-2}	2.17 (1.06 - 4.43)
MOD (4.7%)	SIDD (3.8%)	5.1×10^{-1}	1.24 (0.71 - 2.25)
MOD (4.7%)	MARD (1.8%)	1.9×10^{-4}	2.69 (1.53 - 4.94)

Subjects with kidney function in Stages 1 and 2 were considered normal, and those with kidney function classified as Stages 3A, 3B, and 4 were considered as having chronic kidney disease (CKD). Results include the p-values from Fisher's Exact Test alongside the odds ratio (OR) for CKD and 95% confidence intervals (CI).

Table S3: Comparison of kidney function between "Normal" and "T2D subtypes".

Category (% CKD)		*p*-value	Odds ratio (95% CI)
SIDD (3.8%)	Normal (0.1%)	5.0×10^{-16}	23.03 (11.01 – 49.05)
SIRD (11.3%)	Normal (0.1%)	2.2×10^{-16}	73.12 (31.18 – 169.59)
MOD (4.7%)	Normal (0.1%)	2.2×10^{-16}	28.60 (15.77 – 54.96)
MARD (1.8%)	Normal (0.1%)	3.4×10^{-10}	10.60 (5.029 – 22.68)

Subjects with kidney function in Stages 1 and 2 were considered normal, and those with kidney function classified as Stages 3A, 3B, and 4 were considered as having chronic kidney disease (CKD). Results include the p-values from Fisher's Exact Test alongside the odds ratio (OR) for CKD and 95% confidence intervals (CI).

Table S4: Comparison of kidney function between T2D subtypes based on ANDIS coordinates classification.

Subtypes (%CKD)		*p*-value	Odds ratio (95% CI)
SIRD (7.9%)	SIDD (3.9%)	2.27×10^{-2}	2.09 (1.06 - 4.18)
SIRD (7.9%)	MOD (3.0%)	1.31×10^{-3}	2.75 (1.45 -5.24)
SIRD (7.9%)	MARD (3.3%)	1.12×10^{-3}	2.50 (1.42 - 4.34)
SIDD (3.9%)	MARD (3.3%)	5.5×10^{-1}	1.19 (0.63 - 2.15)
MOD (3.0%)	SIDD (3.9%)	4.1×10^{-1}	0.76 (0.38 - 1.52)
MOD (3.0%)	MARD (3.3%)	7.9×10^{-1}	0.91 (0.51 - 1.58)

Subjects with kidney function in Stages 1 and 2 were considered normal, and those with kidney function classified as Stages 3A, 3B, and 4 were considered as having chronic kidney disease (CKD). Results include the p-values from Fisher's Exact Test alongside the odds ratio (OR) for CKD and 95% confidence intervals (CI).

Table S5: Comparison of MASLD between different T2D subtypes.

Subtypes (%MASLD)		*p*-value	Odds ratio (95% CI)
SIRD	SIDD	8.82×10^{-2}	0.33 (0.06 - 1.11)
SIRD	MOD	2.66×10^{-1}	2.11 (0.66 - 10.67)
SIRD	MARD	5.82×10^{-4}	5.45 (1.77 - 27.24)
SIDD	MARD	1.43×10^{-3}	1.85 (1.24 - 2.81)
MOD	SIDD	1.34×10^{-1}	0.71 (0.45 - 1.13)
MOD	MARD	3.91×10^{-9}	2.59 (1.85 - 3.66)

Results include the *p*-values from Fisher's Exact Test, the odds ratio (OR), and 95% confidence intervals (CI).

Table S6: Comparison of kidney function between the 24 MODY individuals in QBB.

Cohort		SIDD	SIRD	MOD	MARD
**Sample Size (N)**		1 (4.3%)	-	5 (21.7%)	17 (73.9%)
**Kidney function**	**Stage 1 or 2 (Normal)**	-	-	4 (17.3%)	17 (73.9%)
	**Stage 3A, 3B, 4 (CKD)**	1 (4.3%)	-	1 (4.3%)	-

The number of individuals and percentage are listed. The stages of kidney function: Stage 1 or 2 are considered (Normal), while the stages of kidney function: Stage 3A, 3B, or 4 are considered chronic kidney disease (CKD). *One individual with MODY, which did not cluster due to a missing variable, is not included and had a normal kidney function (stage 1).

Table S7: QBB coordinates for T2D subtypes.

Subtypes	Age of diagnosis	BMI	HbA1C	HOMA2B	HOMA2IR
SIDD	-0.3152362	0.01740378	1.5814537	-0.64733910	0.09810316
SIRD	-0.4364714	0.30310395	0.2788862	2.62034740	3.20065965
MOD	0.8288395	0.28959621	-0.2428693	-0.05797478	-0.14058439
MARD	-0.6975420	-0.35781997	-0.5527749	0.05487771	-0.27194420
